# Modeling the tumor immune microenvironment for drug discovery using 3D culture

**DOI:** 10.1063/5.0030693

**Published:** 2021-02-02

**Authors:** Joanna Y. Lee, Ovijit Chaudhuri

**Affiliations:** 1 Department of Biochemical and Cellular Pharmacology, Genentech, South San Francisco, California 94080, USA; 2 Department of Mechanical Engineering, Stanford University, Stanford, California 94305, USA

## Abstract

A few decades ago, the notion that a patient's own immune system could recognize and eliminate tumor cells was highly controversial; now, it is the basis for a thriving new field of cancer research, cancer immunology. With these new immune-based cancer treatments come the need for new complex preclinical models to assess their efficacy. Traditional therapeutics have often targeted the intrinsic growth of cancer cells and could, thus, be modeled with 2D monoculture. However, the next generation of therapeutics necessitates significantly greater complexity to model the ability of immune cells to infiltrate, recognize, and eliminate tumor cells. Modeling the physical and chemical barriers to immune infiltration requires consideration of extracellular matrix composition, architecture, and mechanobiology in addition to interactions between multiple cell types. Here, we give an overview of the unique properties of the tumor immune microenvironment, the challenges of creating physiologically relevant 3D culture models for drug discovery, and a perspective on future opportunities to meet this significant challenge.

## THE TUMOR IMMUNE MICROENVIRONMENT

A tumor starts with a single cancer cell. Due to genomic instability, this one cell proliferates into a population of heterogenous cancer cells, which begin remodeling their environment with the help of neighboring fibroblasts.^1^ Cancer cells make up only a small subset of the tumor, with newly formed extracellular matrix (ECM) as a main contributor to tumor mass, giving the tumor its characteristic tissue stiffness and density.^2,3^ Cancer cells can secrete small amounts of ECM; however, fibroblasts are predominantly responsible for ECM deposition and organization.^4,5^ Fibroblasts residing in the tumor microenvironment (TME) are broadly termed cancer associated fibroblasts (CAFs).^6^ CAFs contribute strongly to building the TME, with the degree of ECM stiffening and immune infiltration highly correlating with disease prognosis.^7^

**FIG. 1.**
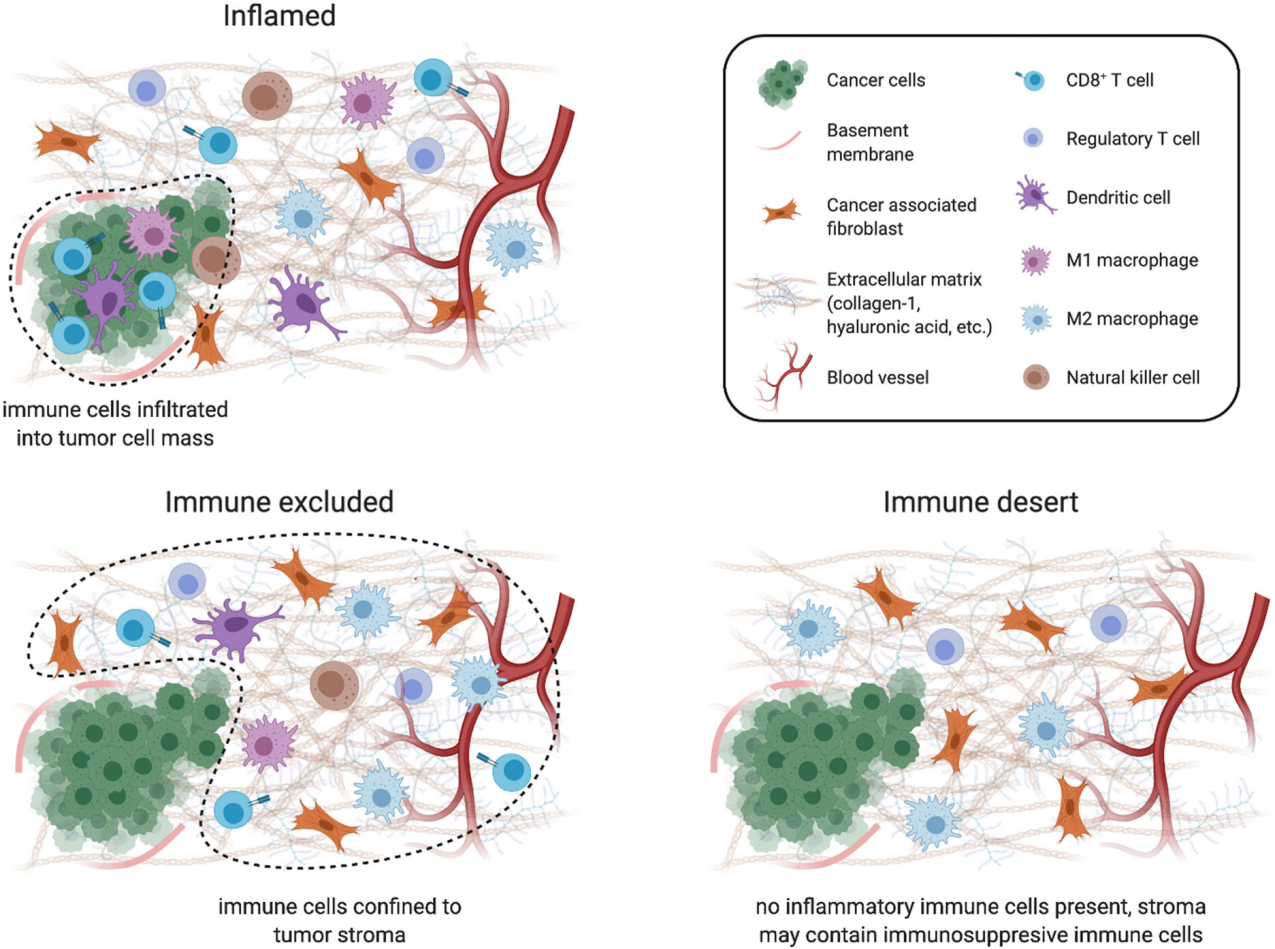
Cells associated with different cancer immune landscapes. Schematic depicting various cancer immune environments and the cells associated with them. Created using BioRender.com.

There are three known cancer-immune landscapes (Fig. 1): (1) inflamed, (2) immune excluded, and (3) immune desert.^8^ In an inflamed tumor, antitumor immune cells infiltrate the tumor stroma and come into direct contact with cancer cells. In this scenario, cancer cells can still evade immune cell killing through mechanisms such as upregulation of checkpoint inhibitors (e.g., PD-L1) on the cancer cell surface. However, not all inflamed tumors respond to checkpoint inhibition therapy, demonstrating that cancer cells use additional strategies to evade immune targeting in inflamed tumors. In an immune excluded tumor, immune cells are present, but confined to the stroma, residing at the border of the cancer cell mass but failing to infiltrate into it. Immune-excluded tumors are typically unresponsive to checkpoint inhibitor treatments such as anti-PD-L1, as enabling cancer cell recognition is worthless if T cells cannot physically come into contact with cancer cells. Finally, immune desert tumors are devoid of antitumor immune cells from both the cancer cells and stroma. While immune cells may be found in these tumors, they are typically regulatory immune cells that are recruited by tumors to suppress antitumor immunity. Immune deserts show a distinct lack of inflammatory signaling with little to no CD8^+^ T cells. This may be attributed to T cell exhaustion or the lack of necessary T cell priming and activation.^8^

Tumors suppress immunity by acting directly on the local immune cell repertoire and indirectly by creating a hostile environment that inhibits immune function. This includes secretion of immunosuppressive factors (e.g., TGF-β),^9^ lack of CD8^+^ T cell attracting chemokines (e.g., CCL5),^10^ increasingly fibrotic ECM,^11^ and depletion of oxygen and nutrients.^12–14^

## TUMOR MICROENVIRONMENT CELL TYPES

### Cancer-associated fibroblasts (CAFs)

CAFs are known for their characteristically pro-tumorigenic phenotypes, which include increased ECM deposition and immune suppression.^15^ While it has been conclusively shown that CAFs arise from local microenvironment fibroblasts, rather than recruited precursors as previously proposed,^1^ how cancer cells convert fibroblasts into CAFs is still unknown.^16^ Additionally, CAFs are difficult to define as they represent a heterogeneous population.^17,18^ Studies show that cancer cells metastasized to a new tissue site produce mostly ECM regulators and secreted factors, while the majority of structural ECM proteins, and thus tumor mass, are instead produced by stromal cells native to the tissue site.^5^ This suggests that fibroblasts may be converted to CAFs through paracrine signaling by cancer cells. Interestingly, total elimination of fibroblasts resulted in worse outcomes, suggesting that fibroblasts play dual roles such as preventing and promoting malignancy.^16,19,20^ This is supported by single-cell RNA-sequencing (scRNA-seq) studies showing that different subsets of CAFs with distinct functions exist within a single tumor.^17,18^ While total elimination of fibroblasts is known to be detrimental, modulating fibroblast signaling can significantly reduce their pro-tumorigenic functions.^21^

CAFs create a chemical barrier to immune infiltration by secreting signaling proteins and create a physical barrier by depositing and cross-linking significant quantities of ECM components, such as collagen-1 (col-1) and hyaluronic acid (HA).^2,22^ Reduced matrix pore sizes associated with denser ECM physically limit the ability of cells to migrate through the matrix. However, this is a fine balance, as increased ECM deposition-associated increased microenvironment stiffness can also promote cancer cell proliferation and migration through mechanotransduction.^23,24^ Additionally, increased extracellular concentrations of ECM components like col-1 and HA, which are biologically active signaling ligands, enhance cancer cell aggressiveness.^25^ In addition to their roles in generating ECM, CAFs influence the TME by forming biochemical gradients that modulate the immune landscape.^26^ This can be accomplished by modulating the concentration of chemokines, signaling proteins that attract white blood cells.^27^ Chemokines required for recruitment of antitumor immune cells, such as CD8^+^ T cells, are decreased, while chemokines required for recruitment of immunosuppressive immune cells, such as T regulatory (Treg) cells and suppressive myeloid cells, are increased. CAFs also release signaling proteins and growth factors to promote cancer cell proliferation.^27^

Mediating TME metabolism is another area that CAFs play important tumor supportive roles.^13,14^ The stroma is the ECM-filled region of tissue that separates and supports epithelial linings. The stroma houses not only fibroblasts but also blood vessels and immune cells. Angiogenesis, the generation of new blood vessels from existing vasculature, is required to supply oxygen and nutrients to the tumor.^28^ CAFs aid in angiogenesis through the production of chemokines such as CXCL12.^29^ Angiogenesis inhibitors, such as anti-Vascular Endothelial Growth Factor (VEGF), have been used as a way to restrict the tumor size by cutting off their supply of oxygen and nutrients; tumors are limited to 1–2 mm^3^ in the absence of new blood vessels.^28^ However, promoting a hypoxic, nutrient-poor environment is also detrimental to antitumor immune cells.^12^ Immune cells are highly sensitive to hypoxia and glucose deprivation, while cancer cells are less sensitive and can rely on CAFs to provide limiting nutrients.^12–14^ CAF-promoted angiogenesis could also decrease metastatic potential. Given that hypoxia drives increased ECM production, which drives cancer cell invasion, it is possible that increased angiogenesis and subsequent normoxia inhibit propensity for cancer cell invasion.^30^

### Immune cells

*In vitro* studies that comprehensively include all human immune cell types generally use peripheral blood mononuclear cells (PBMCs). PBMCs are separated from red blood cells and other elements of whole blood by density gradient centrifugation and include T cells (40%–60%), B cells (5%–10%), Natural Killer (NK) cells (10%–30%), monocytes (5%–10%), and dendritic cells (DCs; 1%–2%).^31^ For isolation of specific immune cell subpopulations, PBMCs can be separated by cell surface markers unique to each cell type.

T cells have been a major focus of cancer immunology, specifically cytotoxic CD8^+^ T cells. CD8^+^ T cells seek and destroy cancer cells through recognition of antigens presented on the cancer cell surface.^32–34^ While professional antigen-presenting cells (APCs) use major histocompatibility complex (MHC) class II molecules to present antigens from extracellular proteins, most cells in the body are not professional APCs and, instead, present peptides derived from intracellular proteins on MHC class I molecules. In cancer cells, the antigens presented are typically proteins from mutated cancer genes, giving them enough contrast from normal proteins to be recognized by the T cell receptor (TCR) as nonself. Effective T cell stimulation also requires binding of surface receptors such as CD28, typically by APCs, which provides an additional barrier to aberrant T cell activation. *In vitro* T cell activation can be achieved by incubating with CD3 and CD28 antibodies, as anti-CD3 binding activates the TCR and anti-CD28 binding provides co-stimulation.^32–34^ Transgenic mouse models also facilitate *in vitro* T cell activation, for example, using the ovalbumin (OVA) system. Epithelial cells are isolated from transgenic mice expressing chicken ovalbumin and paired with T cells from transgenic mice expressing an OVA-specific TCR, enabling antigen binding and T cell activation.^35^

Checkpoint receptors on the T cell surface act as a further safety mechanism, facilitating T cell exhaustion to limit immune-mediated tissue damage during persistent infection; however, this process is readily hijacked by tumors.^33,34^ The two best studied immune checkpoints are cytotoxic T-lymphocyte protein 4 (CTLA4) and programmed cell death protein 1 (PD-1).^36^ CTLA4 is a receptor expressed on tumor cells, regulatory T cells, and exhausted T cells that negatively regulate CD8^+^ T cells. CTLA4 outcompetes CD28 for binding to its APC presented co-stimulation ligands, CD80 and CD86, by binding them with much higher affinity.^37^ PD-1 is a negative regulator that is expressed on stimulated T cells.^38^ Binding of PD-1 to its ligands PD-L1 or PD-L2 disrupts intracellular TCR/CD28 signaling, inhibiting T cell activity. PD-L1 and PD-L2 are normally expressed by APCs and various nonmalignant tissues.^39^ Tumor cells evade immune detection by exploiting vulnerabilities in the multistep process of T cell activation at various stages, including downregulating MHC class I antigen presentation, expressing CTLA4, or upregulating PD-L1 expression. As such, checkpoint inhibitor therapies, inhibiting CTLA4 and PD-L1/PD-1, were revolutionary cancer immunotherapies (CITs) as they restored the ability of CD8^+^ T cells to recognize and kill cancer cells.^36^

Treg cells, on the other hand, play natural immunosuppressive roles and are important regulators of immune tolerance. By decreasing the ability of DCs and macrophages to activate CD8^+^ T cells, Treg cells normally safeguard against overextended immune activation and subsequent autoimmunity. Accumulation of Treg cells is a common feature of tumors and, thus, an attractive therapeutic target. Given that the Treg cell mechanism of action depends on DCs and macrophages, these other cell types are also being pursued as therapeutic targets for releasing immune suppression.^40^

DCs are professional APCs. These cells engulf foreign material, process that material into peptides known as antigens, and present them on MHC class II receptors on the cell surface. The antigen-bound MHC class II complex is recognized and bound by TCRs on the T cell surface, which is required for T cell activation. DCs are especially important in T cell activation as they present a wide range of antigens and regulate T cell proliferation and function.^41^ DC populations are kept low in tumors by the lack of chemokines required for their recruitment, with, instead, the presence of immune suppressing cells such as Tregs, monocytes, and macrophages promoted by an abundance of cancer cell-secreted chemokines (e.g., CXCL1, CCL2, and CCL20).^41,42^ While DCs constitute a minute fraction of the total PBMC population and overall tumor content, their number positively correlates with cancer patient survival.^43,44^ As such, boosting DC maturation, expansion, and infiltration in tumors is being explored as a potential avenue for increasing antitumor immunity.^45^

Monocytes are large white blood cells that can differentiate into either macrophages or a class of DCs, called monocyte-derived DCs.^46^ Macrophages are the most prevalent immune cells in solid tumors and possess a plasticity that allows their polarization into one of the two types: M1 and M2. M1 macrophages normally act as the first line of defense against pathogens and play important roles in wound healing. Like DCs, macrophages are APCs that phagocytose foreign cells and activate CD8^+^ T cells through antigen presentation.^46^ Tumor-associated macrophages (TAMs), however, are generally M2-type macrophages that inhibit inflammation, which suppresses immunity to promote cancer progression.^40^ Given that tumors behave similar to “wounds that do not heal,”^47,48^ they readily co-opt the immunosuppressive wound healing activities of M2 macrophages that normally prevent tissue damage from prolonged immune activation.^46^ TAMs can be recruited into tumors by chemokines such as CCL2, CCL3, CCL4, CXCL12, IL-6, and IL-1β. Some of these chemokines bind and activate integrins, specifically α4β1 integrin, promoting TAM migration into tumors.^46^ Once there, TAMs secrete growth factors and cytokines that suppress T cell activation and promote ECM remodeling.^49,50^ While depletion of macrophages seems a logical therapeutic approach, loss of all macrophages can lead to severe side effects. As macrophages have both immune suppressing and activating functions, pharmacologically encouraging TAMs toward their immune activating, M1, antitumor state is more desirable.^50^

NK cells are lymphocytes that directly eliminate foreign cells by releasing cytotoxic granules and do not require multistep activation like CD8^+^ T cells. They can recognize cells with decreased MHC class I expression, which includes cancer cells.^51^ NK cells also recognize certain ligands that are overexpressed on cancer cells and cells undergoing infection, cellular stress, or DNA damage.^44,52^ Indirectly, in a process known as antibody-dependent cellular cytotoxicity (ADCC), NK cells can destroy tumor cells by binding antibody-coated tumor cells using their CD16 receptors, triggering release of their cytotoxic granules.^44^ While they do not directly activate T cells, NK cells contribute to T cell function by secreting cytokines that activate DCs and stimulating recruitment of DCs into the TME.^50^ Unsurprisingly, NK cell activity and tumor infiltration are associated with better outcome, and some tumors are devoid of NK cells.^53^ In addition to strategies to increase endogenous NK cell activity and infiltration *in vivo*, other therapies involve *ex vivo* activation, expansion, and genetic modification of NK cells for infusion into patients.^54^

## 3D IMMUNE CELL MIGRATION AND MECHANOBIOLOGY

Immune cell trafficking is a key part of immune function and activity in the tumor microenvironment and, therefore, must be a key consideration in 3D assays. Tissue-resident macrophages patrol their local microenvironment to find and remove apoptotic cells and cellular remnants, respond to infections, and contribute to tissue remodeling. DCs travel to and from the lymphoid organs, such as the lymph nodes, to nonlymphoid organs where they migrate and search for pathogens. T-cells migrate to interact with APCs and to target tissues in order to orchestrate the local immune response. With such a critical role in immune cell function, immune cell migration has been a focus of increasing study.

One of the broad ideas emerging from studies of immune cell migration is that immune cell migration differs from the migration of typical adherent cells such as epithelial cells, fibroblasts, and cancer cells. The key force generating modules mediating migration of adherent cells have been identified. Actin polymerization at the leading edge generates force and can propel the front of the cell forward when coupled to integrin-based adhesions of sufficient strength.^55^ Myosin-based contractility can release adhesions and also squeeze the nucleus and cell body forward. Differences in osmotic pressure can also lead to water and ion flow that drive cell displacement.^56,57^ How these modules interact to drive migration in 3D microenvironments is complex and varies depending on the context, but basic rules have been identified.^58^ When pore sizes in the matrix are sufficiently large, cells can migrate using various modes of migration including both mesenchymal modes, involving spread morphologies, and ameboid modes, involving more rounded morphologies. As the matrix pore size falls below around 3 *μ*m, the nucleus of adherent cells becomes a barrier to migration, as cells are unable to deform the stiff nucleus through small pores.^56,59^ These microenvironments are typically referred to as confining microenvironments.^60,61^ Adherent cells can overcome this barrier by utilizing proteases,^56^ typically matrix metalloproteinases (MMPs), to degrade the matrix, applying mechanical force to dilate pores in the matrix if the matrix is malleable or mechanically plastic,^62^ or some combination of both. While the same force-generating modules are implicated in immune cells, there are some key distinctions in their migration behaviors. Generally, immune cells are able to migrate through much smaller pore sizes due to a softer nucleus and rely less on adhesions to the matrix and matrix degradation. Immune cells can also migrate at high speeds in 3D, commonly migrating at speeds ranging from 1.0 to 10.0 *μ*m/min, while adherent cells migrate commonly at speeds ranging from 0.1 to 1.0 *μ*m/min.^63^ However, each type of immune cell exhibits its own distinct features of migration.

Macrophages exhibit the most similarity to adherent cells in their migration. Macrophages can migrate in an ameboid manner, where they take on a rounded morphology and migrate at higher speeds (approaching 1.0 *μ*m/min) independent of proteolytic activity and minimally relying on adhesions.^64^ Alternatively, they can migrate in a mesenchymal manner, where they migrate at a much slower rate (∼0.1 *μ*m/min) and rely upon matrix adhesions and matrix degradation. Fibrillar collagen matrices promote ameboid migration, while nanoporous collagen matrices or reconstituted basement membrane (rBM) matrices promote mesenchymal migration.^65^ In the mesenchymal mode of migration, macrophages extend actin-rich protrusive structures known as podosomes, which degrade matrix using MT1-MMP, apply mechanical force to the compact matrix, and engulf matrix, thereby generating migration paths.^66^ M1 type or pro-inflammatory macrophages have been found to be less motile than M2 or anti-inflammatory, macrophages.^67^

DCs have remarkable migration characteristics that are not found in adherent cells. Importantly, DCs can migrate independent of integrin-matrix adhesions in confining 3D matrices, as deletion of integrins does not impede their migration *in vivo.*^68^ Integrin-independent migration of DCs relies upon actin network expansion that drives the leading edge into openings. In this mode, instead of pushing off of adhesions, DCs utilize topographical features of the substrate to propel themselves forward.^69^ Interestingly, DCs can switch between integrin-independent and integrin-dependent migration modes to maintain migration velocity.^66,70^ In contrast to macrophages, it is thought that DCs do not rely on proteases for migration path generation.^71^ DCs can squeeze through narrow gaps with diameters approaching 1 *μ*m, with myosin-mediated contractility and disruption of the nuclear lamina through Arp2/3-driven actin facilitating nuclear deformation.^72^ The Rho GTPase CDC42 plays a major role in orchestrating migration of DCs by directing activity of the Arp2/3 complex to initiate actin network polymerization at the leading edge, with the activity of CDC42, in turn, mediated by the Rho GEF Dock8.^73^ During DC migration, the microtubule organizing center (MTOC) is located behind the nucleus in contrast to mesenchymal cells, and this organization facilitates the use of the nucleus by DCs to sense the path of least resistance in complex 3D microenvironments.^74^

T cells share many similarities in migration characteristics with DCs but have their own unique characteristics. In adhesion-dependent migration, T cells utilize integrins such as αvβ1 to bind RGD peptides commonly found in collagen and fibronectin,^67^ as well as leukocyte-specific adhesion receptor α_L_β_2_ integrin (LFA-1) to bind ICAM-1.^75,76^ Similar to DCs, T cells can also migrate independent of adhesions. However, while DCs can migrate in even smooth channels, T cells require some texture in the channels to push off,^55^ perhaps because T cells are rounded and lack the dendrites characteristic of DCs. Interestingly, T cell activation is significantly enhanced by substrate stiffness, potentially by mimicking the opposing forces exerted during APC binding.^77^

While key insight into immune cell migration has been established, a number of outstanding questions remain. There is now tremendous evidence of how physical properties of the ECM, including matrix architecture, stiffness, and mechanical plasticity, impact adherent cell migration in 3D.^78–82^ As the TME often exhibits striking differences in each of these characteristics relative to normal microenvironments, these findings are thought to be critical to understanding cancer pathogenesis. However, very little is known about how these matrix properties do, or do not, impact the migration of immune cells.

Another important question lies in the potential mechanosensitivity of immune cells. Adherent cells are well known to sense and respond to mechanical properties of the extracellular matrix, with changes in extracellular matrix stiffness and viscoelasticity regulating cell spreading, proliferation, gene expression and the epigenome, cancer progression, and stem cell fate.^77^ Mechanistically, cells sense matrix properties, in part, by gauging resistance to contractile forces applied to the matrix through integrin-based adhesions. As immune cells form adhesions with matrices and can apply force to matrices through these adhesions, it might be similarly reasoned that matrix properties should strongly impact the functional activity of immune cells. However, for DCs and T-cells that travel through numerous tissues that have a wide range of mechanical properties, it might also be expected that these cells should be relatively “immune” to matrix stiffness and viscoelasticity. Some studies have shown that macrophage and DC phenotypes are impacted by substrate stiffness.^83^ However, many of these studies were conducted in 2D culture, while immune cells function in 3D microenvironments. Importantly, mechanotransduction in adherent cells is known to be strongly dependent on culture dimensionality. For example, while the Yes-Associated Protein (YAP) transcriptional regulator is considered to be the universal mechanotransducer from 2D studies, YAP does not mediate mechanotransduction in a 3D culture model of breast cancer nor is it implicated from analysis of breast cancer patient samples.^75^ Therefore, in order to form appropriate 3D culture models of the TME, the mechanosensitivity of immune cells must also be elucidated. Several recent studies have started to make progress along this front. In one study, increased collagen density was found to downregulate cytotoxic activities and upregulate regulatory markers of T cells,^84^ impairing the ability of the cells to kill cancer cells. As mentioned previously, another study explored the impact of matrix stiffness on T cell activation using microporous alginate scaffolds as 3D culture matrices and found that increased stiffness facilitated T-cell interactions with APCs and T-cell activation.^75^ Much more studies are needed to gain deeper insight into how the mechanical properties of the TME modulate immune cell activity.

## ASSAYS FOR CANCER IMMUNOTHERAPIES

Examining the 96.6% failure rate of oncology drugs in clinical trial, roughly half fail to pass phase I safety studies.^85,86^ Of the remaining clinical candidates who progress into phase II and phase III, about half fail due to the lack of efficacy. This suggests that 2D culture with human cells and preclinical testing with animals fail to recapitulate important features of human biology that impact both the safety and the efficacy of a drug.

**FIG. 2.**
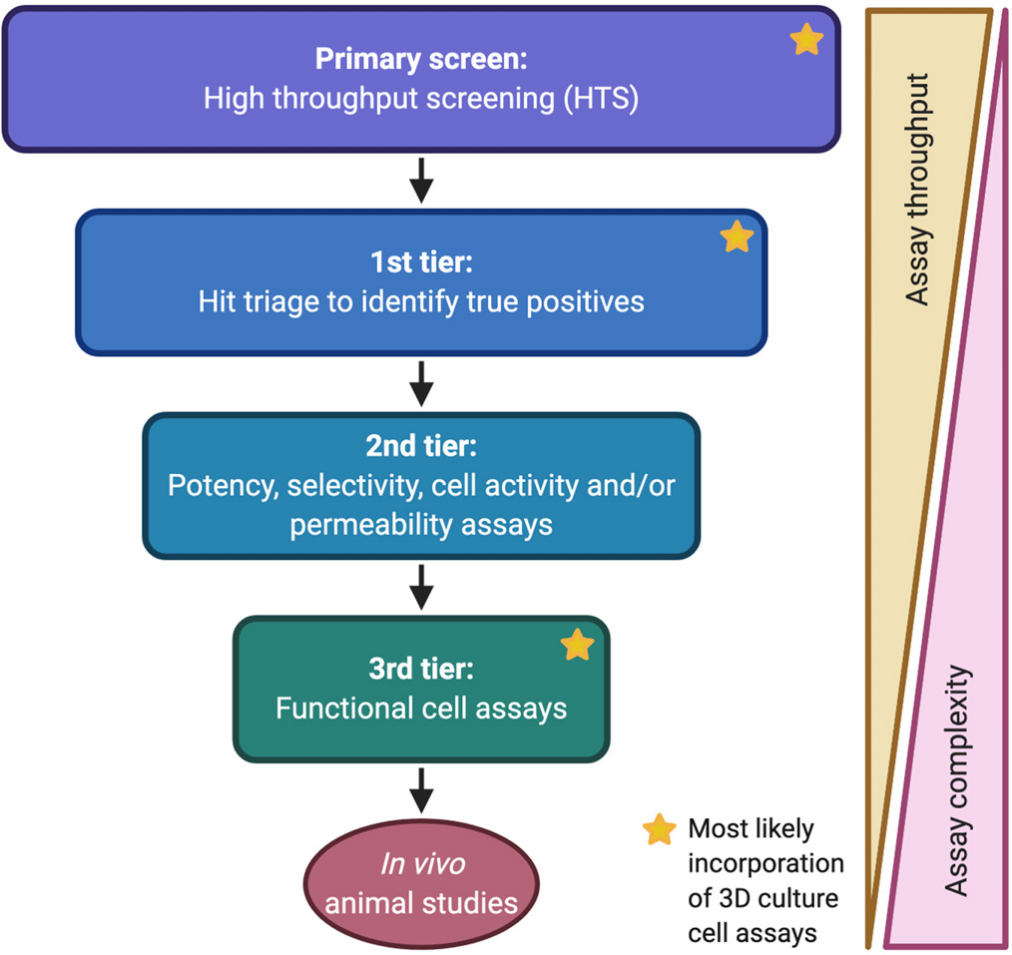
Drug discovery assay cascade. Example assay cascade for drug discovery and where 3D culture assays could be included. Created using BioRender.com.

To propose how 3D cultures can be incorporated into drug discovery, it is critical to first understand where they would fit in an assay cascade (Fig. 2). In drug discovery, an assay cascade is the progressive set of experiments potential drugs are funneled through to find the ones with the best chance of *in vivo* success.^84^ In the target-focused approach to drug discovery, there is a known therapeutic target, e.g., a protein that is known to suppress cancer immunity. Alternatively, the phenotypic screening approach is target agnostic,^87,88^ instead using an intended phenotype as the starting point and screening for compounds that induce that phenotype, e.g., T cell infiltration into the tumor stroma. Whether target-focused or phenotypic, the first step is to conduct a screen using a library of compounds to identify molecules that bind the target or induce the desired phenotype. Thousands or millions of compounds go into the primary screening assay. Since primary screening assays require high throughput, they are typically biochemical assays or simple cell-based assays with luminescence or fluorescence readouts. A phenotypic screen for activators of antitumor immunity using a 3D culture assay could be very powerful; however, reaching the necessary throughput with a physiologically relevant 3D culture setup is extremely challenging. Following the primary screen, a first-tier assay, or assays, takes the hits identified in the primary screening assay and uses either biochemical or cell assays to triage true positives;^88^ these true positives provide the substrate for iterative optimization to generate a potent and selective drug. Even in 2D culture, cell assays have lower throughput than their biochemical counterparts, and sometimes, the throughput is prohibitive for unoptimized compounds. In cell assays, compounds are required to cross a cell membrane and potentially compete with intracellular proteins for binding to the target, while exhibiting sufficient selectivity for the target to avoid confounding activities that may mask the true-positive effect. True positives can be optimized by medicinal chemistry or antibody affinity maturation and then evaluated in the assay cascade. Only the subset shown to be active makes it into the next tier of the cascade, and the next, with the compounds with the best potency, selectivity, and functional activity making it through the entire cascade.

In the second tier of the assay cascade, more biochemical or cell-based assays follow, identifying the compounds with highest potency and specificity to the target of interest and eliminating promiscuous compounds that would cause off-target effects. Second tier assays may also include cell assays that aid in ascertaining compound permeability and in finding whether it is active in cells (i.e., engagement of the target signaling pathway). Functional cell activity of CITs is much more difficult to define in a 2D setting (e.g., immune infiltration). To faithfully model whether a compound enables immune cells to overcome the physical and chemical barriers to antitumor immunity, e.g., fibrotic ECM, hypoxia, 3D matrix signaling, lack of nutrients/chemokines, etc., a culture would require not only multiple cell types but also recapitulation of those physical 3D barriers and ECM architecture. Drugs nominated as clinical candidates are only as good as the assay cascades used to identify them. The introduction of physiologically relevant 3D culture assays could significantly improve our ability to progress candidates with a higher chance of *in vivo* efficacy;^89^ however, this hinges on generating effective 3D culture models, which requires not only technical advancement but also better understanding of the biological processes driving the TME.

While the lack of reliable 3D culture assays for cancer immunology has not prevented the discovery and approval of currently available CITs (e.g., checkpoint inhibitors), increasing the speed and success with which new CITs are approved would be highly impactful. Checkpoint inhibitors were a major breakthrough in our understanding of cancer immunology and, because they target a characteristic of inflamed tumors, could be modeled by combining T cells and cancer cells in 2D culture. However, given that checkpoint inhibition does not affect the physical separation between T cells from tumor cells, as seen in noninflamed immune excluded and desert phenotypes,^8^ they offer clinical benefit to only a small subset of patients, many of whom eventually become resistant and relapse.^89–91^ As most tumors tend to be noninflamed, or cold (e.g., pancreatic, breast, colon, etc.), there has been significant interest in finding treatments that specifically turn cold tumors hot or inflamed.^85,86^ This requires 3D culture assays that encompass the relevant, complex barriers to immune infiltration so that we can assess the ability of immune cells to overcome them in the presence of candidate CITs. The lack of reliable 3D culture models makes it necessary in most cases to go straight from 2D functional cell assays into preclinical animal models.^91^ While animal studies have the advantage of taking into account the whole organism, preclinical testing for even a single drug is resource and labor intensive; a well-controlled study requires replicates with several doses and varying dosing schedules. As such, only the seemingly best performing compounds that make it through the assay cascade are selected for preclinical testing. The best performing compounds in a 2D cell context may not be the best in a 3D cell context. In addition, there are significant limitations to relying on preclinical mouse models to ascertain efficacy of CITs.^93–95^ Preclinical cancer models typically involve mice inoculated with human tumors or genetically engineered mouse models (GEMMs), which form tumors *de novo*. Mice are often immunocompromised due to inbreeding and have a significantly different immune repertoire from human, which can lead to confounding results when testing CITs on human tumors in mice. While GEMMs form syngeneic tumors, these tumor-bearing mice are generated through introduction of germline genetic mutations, thus forming stable cancers that often lack the genomic instability of human tumors required to respond to CITs.^91,95,96^ Therefore, well-designed 3D culture models using human cells could potentially be more predictive of clinical efficacy for CITs than preclinical mouse models.

The first known cancer immunotherapy was Coley's toxin, a mixture of bacteria that was injected into more than a thousand cancer patients to induce an antitumor response.^93,97^ The field of cancer immunology has evolved significantly, with modern CITs falling broadly into five categories: checkpoint inhibitors, adoptive T cell transfer therapy, monoclonal antibodies, cancer vaccines, and immune modulators.^92–94^ 3D culture systems that faithfully recapitulate the TME for screening new therapies are currently the holy grail of drug discovery.^98^ This task is immensely challenging from both a technical and biological standpoint: how do we build a model of a cold tumor when we barely understand the mechanisms that make it cold? To make building such a model even more challenging, there are currently no positive controls to aid in assay development; there are no approved drugs yet that can activate immune cells in cold tumors.

While the focus of this Perspective is on the future of complex, physiologically relevant 3D culture assays, it is worth noting that many important cancer immunology discoveries have been made using 2D culture. These include assays for immune activity such as proliferation assays, cytokine assays, and MHC expression assays.^32^ More complex 2D culture assays include CD8^+^ T cell killing assays of cancer cells, which generally involve cells isolated from mouse models.^99^ While the use of human cells would be ideal, this is a significant challenge as immune cells would have to originate from the same organism as the cancer cells in the assay to avoid indiscriminate killing by immune rejection, i.e., antigens derived from cells of another organism being recognized by CD8^+^ T cells as foreign. Patient samples are also limiting, rarely yielding enough material for well-controlled studies. Human PBMCs are also an option; however, very few T cells isolated from healthy donor PBMCs possess the TCRs specific to a cancer cell antigen, as expansion of these cells normally occurs in a cancer patient after DC-mediated T cell stimulation and proliferation. However, recent studies demonstrate that cancer antigen-specific T cells may be expanded *in vitro*, in a DC-dependent manner, by culturing human tumor organoids with PBMCs from the same patient.^100,101^

Given the multitude of immune cell types, diverse mechanisms that cancer cells employ to evade immune cells, and highly specific mechanisms of action of modern immunotherapies, the challenge in building a physiologically relevant 3D culture model of the tumor immune microenvironment is clear: there is no one-size-fits all assay. If a complex assay can only be marketed to a small segment of cancer immunology research, it is much less likely to be developed for sale and distribution. CIT researchers often do not have the equipment or engineering expertise to build specialized 3D culture platforms and scale them for drug discovery. Because of this, broader used assays are often marketed, which often do not capture the relevant TME interactions for the specific CIT being studied, leading to frustration and skepticism for 3D culture assays by researchers. Additionally, functioning TME models that incorporate both cancer cells and fibroblasts are still early in their development, let alone models that incorporate cancer cells, fibroblasts, and immune cells. Looking to the future of 3D culture in drug discovery, it is imperative that 3D culture assays are tailored to the mechanism of action being studied. We discuss here 3D culture models currently being used in CIT and the types of studies they provide the most value. We also propose potential ideas for 3D culture assays that may offer greater physiological relevance for cancer immunology studies and the degree with which they can be scaled for drug discovery.

## EMERGING 3D CULTURE CANCER IMMUNOLOGY ASSAYS

### Scaffold vs nonscaffold cultures

**FIG. 3.**
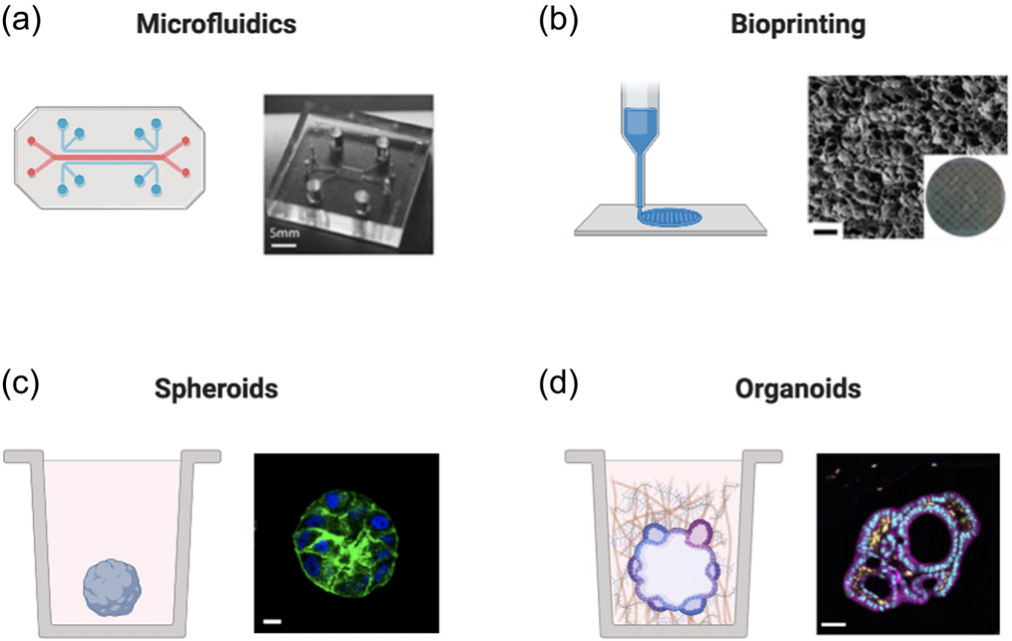
3D culture platforms. Diagram of 2D vs 3D culture platforms currently being explored for cancer immunology studies. (a) Left: microfluidics schematic; right: microfluidics image from Bai *et al.*, Oncotarget **6**, 25295 (2015). Copyright 2015, Authors licensed under a Creative Commons Attribution (CC BY) license. (b) Left: bioprinter schematic; right: scanning electron micrograph of the bioprinted scaffold containing 10% gelatin methacrylate, bar: 100 *μ*m. Reproduced with permission from Zhou *et al.*, ACS Appl. Mater. Interfaces **8**, 44 (2016). Copyright 2016 American Chemical Society. (c) Left: schematic of the spheroid in the well; right: MCF10A spheroid; green: phalloidin, blue: 4′,6-diamidino-2-phenylindole (DAPI), bar: 10 *μ*m. (d) Left: schematic of the organoid in well; right: colorectal adenocarcinoma patient-derived organoids (PDOs); magenta: E-cadherin (E-cad), yellow: Smooth Muscle Actin (SMA), cyan: DAPI, bar: 50 *μ*m. Reproduced with permission from Neal *et al.*, Cell **175**, 7 (2018). Copyright 2018 Elsevier. Created with BioRender.com.

In this section, we discuss a variety of available 3D culture platforms for modeling the tumor immune microenvironment (Fig. 3 and Table I). An important consideration for all these platforms is the presence vs absence and type of ECM. ECM is the 3D scaffold that houses cells *in vivo*. As detailed in the “Tumor Microenvironment Cell Types” and “3D Immune Cell Migration and Mechanotransduction” sections, ECM plays critical roles in creating physical barriers to immune infiltration, facilitating mechanotransduction and cell motility, and presentation of biochemical signaling proteins. Scaffold-based 3D culture models contain animal-derived or synthetic ECM in the form of hydrogels (reviewed in the study by Lee and Chaudhuri, 2017^25^). In the absence of scaffolding, cells can be encouraged to form 3D structures based on the confines of their surroundings, e.g., spheroids in ultra-low attachment wells. These structures tend to be more loosely compacted given the lack of ECM proteins, e.g., laminins, to facilitate ECM anchorage, polarization, and organization. While cells can secrete their own ECM, this is diluted by the surrounding cell culture medium to concentrations insufficient to generate a polymer network except under special circumstances, e.g., hanging drop cultures. The simplest scaffold-based experiments involve mixing cells with reconstituted ECM hydrogels (e.g., Matrigel), depositing the cell-hydrogel mixture into the bottom of a well (e.g., 96- or 24-well plate), allowing the hydrogel to solidify around the cells by placing the plate at 37 °C, and then adding growth medium to the well. Hydrogel domes can also be spotted into the center of a well, reducing the necessary hydrogel volume; this can be especially useful if the cell sample volume is limiting.^102^ However, while sufficiently mimicking the composition of the basement membrane ECM to promote normal epithelial cell behavior is relatively straightforward (e.g., Matrigel), modeling the composition and stiffness of remodeled cancer stroma, the TME, is much more complex; often requiring engineered biomaterials.^25^

**TABLE I.** 2D and 3D culture platforms used to study cancer immunology.

	ECM scaffold	Throughput	Complexity	Particularly useful for studies involving
2D culture	No	High	Low	Cancer cell intrinsic processes (e.g., proliferation, gene expression, etc.)
Microfluidics	Sometimes	Medium	Medium	Oxygen and nutrient gradients, fluid flow, and limited sample volumes
Bioprinting	Yes	Medium	Medium	Defined architecture (e.g., vasculature) and compartmentalized cells or biologics
Spheroids	Sometimes	High	Medium	3D tumor cell organization (e.g., hypoxic core, drug penetration studies, etc.)
Organoids	Yes	Medium	High	Multicellular tumor organization and intracellular interactions

Scaffold-based models are undoubtedly more difficult to build, scale, and analyze. Working with viscous materials in small volumes that solidify quickly is a challenge in itself; having to image through these gels or isolate cells from them adds additional levels of difficulty. The decision on whether a scaffold-based model is necessary depends on the phenotype being studied. For example, CITs designed to promote immune infiltration may require a model with ECM for the immune cells to traverse. Conversely, a CIT promoting killing of cancer cells already in contact with CD8^+^ T cells, e.g., anti-PD-L1, may be tested in a nonscaffold model, as ECM is not part of the target mechanism of action.

When using scaffold-based approaches, the hydrogel used should be thoughtfully chosen as it will have profound effects on cell behavior.^25^ Animal-derived hydrogels are frequently used in drug discovery given their physiological relevance and commercial availability, e.g., reconstituted Basement Membrane matrix (rBM; tradename Matrigel, Cultrex, etc.). While rBM is highly relevant to 3D culture studies investigating the development of epithelial layers normally in contact with the basement membrane, it is less relevant for cancer cultures that *in vivo* have penetrated through the basement membrane and are now surrounded by a col-1 and HA rich stroma.^103^ While the laminins in the basement membrane help keep preinvasive epithelial cells polarized and clustered, col-1 and HA promote malignant progression in invasive cancer cells that now reside in the stroma.^103^ Col-1 may also be a critical component for immune infiltration studies, as col-1 fibers form the tracks that T cells crawl along during inflammation induced migration through the stroma.^73^ While their availability, ease of use, and biological origin are attractive, these animal-derived materials are not without limitations in their physiological relevance, especially without fibroblasts to remodel their architecture, pore size, and mechanical properties *in vitro*. Col-1/rBM hydrogels made *in vitro* are less than 0.1 kPa in typical formulations, while soft tissues *in vivo* range from 0.1–10 kPA in stiffness.^104^ To this end, engineered materials composed of synthetic or non-ECM based hydrogels, e.g., polyethylene glycol (PEG) or alginate, provide more control over mechanical properties and can be conjugated to ligands to enable cell interaction.^25^ However, this bottom-up approach requires that all relevant cancer and immune cell–ECM interactions are known and recapitulated, which is extremely challenging given the many uncertainties in the budding field of cancer immunology.

### Microfluidics

Microfluidics typically involve polydimethylsiloxane (PDMS) or glass-based microdevice chips containing thin channels to house samples of cells.^105^ Channels running parallel to each other containing different cell types can be separated by either a permeable membrane or a ledge to facilitate interaction between compartments.^106^ In the absence of ECM, this is a 2D assay, with cells forming a monolayer on the bottom of the channel. The addition of ECM is possible; however, reaching a physiologically relevant stiffness and concentration of ECM is challenging given the small channels and viscosity of hydrogels. One advantage of microfluidics is that they are highly amenable to imaging as the small volumes force the entire sample to be in close proximity to the bottom of the chip and, therefore, amenable to imaging with high resolution, low working distance microscope objectives. Microfluidic channels also allow for generation of flow and chemical gradients, including oxygen. The channels' small sample volumes make microfluidics especially useful for studies involving very limited sample; conversely, downstream follow-up experiments are difficult as retrieval of the sample from the microfluidic chamber is nontrivial.^105,107^ While the generation of nutrient and oxygen gradients is significant in terms of physiological relevance, whether a cold TME can be mimicked in a microfluidic channel remains to be seen.

While relatively new, there are a growing number of reported studies modeling tumor–immune interactions using microfluidics. Microfluidic studies using col-1 embedded HeLa cells with NK-92 cells showed a reduction of NK cell migration and cancer cell killing compared to 2D culture assays.^108^ Another study involved col-1 embedded liver cancer cell aggregates and showed that monocytes inhibited TCR T cell-mediated cancer cell targeting in a PD-L1/PD-1-dependent manner.^109^ Spatially confined monocytes, endothelial cells, and breast cancer cells embedded in gelatin, a derivative of col-1, showed that T cells exposed to monocytes had improved recruitment associated with increased chemokines.^110^ Additional studies have also modeled DC interaction with cancer cells,^111^ tumor–lymph node interactions,^112^ and macrophages cultured with glioblastoma or lung cancer cells.^113,114^ To the best of our knowledge, there have been no reported studies that include immune cells and CAFs, which are likely essential for generating a cold TME; however, these microfluidics studies have laid the groundwork toward this goal.

### Bioprinting

Bioprinting allows biomaterials and cells to be deposited in intricate, layered patterns to form a 3D structure.^115–118^ This is particularly useful if a defined architecture is required, e.g., vasculature. 3D bioprinting techniques include extrusion-based, inkjet-based, and laser-assisted formats. A key consideration for any bioprinting technique is the hydrogel used, not only its physiological relevance but also its ability to be printed at format-compatible temperatures and timescales. While hydrogels containing cells have been successfully printed into distinct structures to study metastatic seeding,^119^ printing the specific structure of ECM that surrounds cells in the TME is a different challenge. Bioprinting can generate ECM architecture with tunable features at the microscale or above, however not at the sub-micrometer scale—a length scale where polymer fibrillation and the pore size can greatly impact cell fate.^115–118^ To this end, advances in electrospinning enable generation of scaffolds that mimic *in vivo* ECM architecture at the molecular level, with the caveat that cells must be added after scaffold fabrication.^120^ The technology development to print a physiologically relevant ECM first requires fundamental understanding of the relevant features of the cancer immune TME. However, the technology development must still keep pace, and it is notable that 3D culture models containing ECM, cancer cells, fibroblasts or adipocytes, and endothelial cells have been successfully printed.^118^ While 3D bioprinting of lattices has been used to activate and expand T cells,^121^ studies bioprinting the TME with immune cells are still being explored and have yet to be reported, likely due to the relative infancy of this field.^122^

### Spheroids

Spheroids are clusters of cells typically formed from one cell type: cancer cells.^123^ Given that monoculture spheroid plates require little additional effort over traditional 2D plates, it is encouraging how much more complexity can be captured (e.g., hypoxic core and polarization). Indeed, some drug and oncogene effects that cannot be observed in 2D culture are revealed when moving to 3D spheroids.^124^ Heterogenous spheroids containing fibroblasts or immune cells can also be generated; however, this does not necessarily recapitulate the *in vivo* organization. Except in the case of inflamed tumors, immune cells normally exist outside the cancer cell boundary, in the stroma, while cancer cells alone form a densely packed, spheroid-like cluster.^8^ In all cases, inflamed, excluded, and desert fibroblasts also exist in the stroma. As mentioned above, spheroid formation can be achieved in the absence of a hydrogel scaffold, by placing cells in droplets of media, i.e., hanging drop cultures, or ultra-low attachment plates, i.e., spheroid plates, where defined boundaries confine and influence the shape of the cell aggregate. This coupled with the commercial availability of 96- and 384-well ultra-low attachment plates for spheroid generation makes spheroids an easy entry point into 3D culture assays. Spheroids can be generated by simply adding cells and media to the wells of a 96- or 384-well spheroid plate. Spheroid plates also make high throughput screening (HTS) possible. They are amenable to imaging; however, optimization is required as spheroids can still vary in size and shape. Spheroids have been shown to better mimic *in vivo* therapeutic resistance than their 2D culture counterparts.^125^ The addition of transwell filters has also been used to separate immune cells from spheroids as a model for immune cell migration and spheroid killing.^126^

The addition of even small amounts of rBM, e.g., 2% Matrigel or Cultrex, which remain soluble and do not cause gelation, can dramatically improve spheroid compaction, generating a more *in vivo*-like cancer spheroid. This is likely due to the increased concentration of laminins that facilitate cell polarization. Immune cell killing assays can be performed on spheroids by the addition of immune cells. However, this is only relevant for inflamed tumors, as modeling immune excluded or desert tumors requires the barriers to immune infiltration provided by ECM and CAFs; the addition of ECM to spheroid plates is not possible given the ultra-low attachment surface of the wells. Without a surface to adhere to, hydrogels float to the top of the well. While currently limited in their complexity, spheroids have yielded valuable insight into tumor–immune interactions^123^ and incorporating them with other platforms would make them even more powerful.

### Organoids

Organoids are traditionally stem cell cultures that proliferate and self-organize into simplified structures, capturing some organizational and cellular characteristics of the organs they are intended to recapitulate.^127^ Tumor organoids can also be established using malignant mouse or patient tissues.^128,129^ Importantly, organoids can recapitulate the morphology and cytology of the original tumor even after weeks in culture.^129,130^ This facilitates expansion of limiting quantity of patient samples by propagation in 3D culture.^129^ Recently, pancreatic cancer organoids using matched patient tumor cells, CAFs, and immune cells have also been established.^131^ Organoids can also be generated from induced pluripotent stem cells (iPSCs). This would theoretically allow generation of cancer cells, fibroblasts, and immune cells from the same donor. However, organoids are expensive and time intensive to establish. Also, while samples can be expanded in 3D culture, it is unlikely to reach the quantity needed to conduct a high throughput screen (HTS). High variability can also occur between different samples from the same donor (e.g., ratio of stroma to tumor cells, infiltrated area vs noninfiltrated area). Establishing the appropriate readout for an organoid assay presents additional challenges, as end point assays for cell viability or death cannot distinguish different cell types unless they are differentially labeled. Differentially labeling a heterogenous mixture of live primary cells is also nontrivial and requires genetic manipulation if fluorescence labels need to persist over several days or weeks in culture. Image analysis poses another challenge as each organoid will be unique in size, shape, and baseline cell profile. However, if these challenges can be overcome, organoids will almost certainly be profoundly impactful in drug discovery given their exquisite *in vivo* relevance.

## FUTURE DIRECTIONS OF 3D CULTURE CANCER IMMUNOLOGY ASSAYS

The potential for 3D culture models in cancer immunology drug discovery is significant; however, it requires both technological and basic science advances. Extremely powerful 3D culture assay can still be built given our current knowledge, but doing so requires careful identification and recapitulation of TME features that affect the target being investigated, e.g., cell types, ECM composition and stiffness, and hypoxia. Tradeoffs in complexity may be made for better assay reproducibility and throughput, if the phenotype being studied can be replicated in a minimalist system. Here, we discuss assay development directions that could potentially aid in meeting current cancer immunology drug discovery needs.

The most physiologically relevant 3D culture models will require patient samples. The complexity of the TME is currently not understood well enough to confidently reproduce all the important features using a bottom-up approach. For this reason, target agnostic phenotypic screens would be most successful in identifying efficacious drugs using patient samples. As discussed in the Organoid section, this is incredibly challenging given the small amount of sample that can be given by a patient and the lack of a robust readout. However, using expansion of the sample as a 3D organoid culture, this is theoretically possible. Alternatively, pathologically similar samples from different patients could be used in the same screen, if coupled with a strong positive control used to normalize across donors. As for the readout, artificial intelligence platforms for light microscopy have enabled identification of different cell types during live-cell imaging without expression of fluorescent markers and could be highly impactful if made amenable to cells embedded in ECM.^124^ Alternatively, 3D cultures could be subjected to a membrane or DNA stain at the experiment end point, if the technology exists to accurately differentiate cell types based on the size and shape. Additional end point studies also include high-throughput RNA-seq or scRNA-seq. The use of patient-derived tumor organoids in drug screening is extremely attractive and relies largely on technology development to be brought to fruition.

Less physiologically relevant models of the TME that are more amenable to screening could also be achieved without patient-derived organoids. Given that fibroblasts are likely reprogrammed by cancer cells to remodel the TME into the desired immune landscape, it is possible that culturing cancer spheroids with fibroblasts and ECM may enable the fibroblasts to remodel the microenvironment into a TME that inhibits immune infiltration. However, suppressive immune cells may also be required to complete the immune excluded or desert landscape, such as Tregs, monocytes, and macrophages. These cell line-derived cultures have advantages over patient samples in that they are not limited by the cell number and more amenable to genetic manipulation. The ability to express fluorescent tags in each cell type makes imaging readouts far more likely. While this is a more defined approach to building an immune suppressive TME, it has the benefit of not necessarily requiring elucidation of the complex underlying biology and, instead, relies on co-culturing relevant populations of cells under the conditions that approximate the *in vivo* setting.

Even simpler assays of the cancer immune TME could be designed for targets with well-established functions. For example, if a therapeutic is anticipated to activate chemokine production in the TME to recruit immune cells, a 3D co-culture containing only cancer spheroids and CAFs could be generated, without the need for immune cells. After cultures are treated with drugs, chemokine levels could simply be sampled from the supernatant. In this assay format, immune cells could also be included without a much added difficulty. For example, if a therapeutic aims to revive exhausted T cells, a 3D co-culture of cancer spheroids, CAFs, and immune cells could be developed and treated with compounds, and supernatants are assayed for cytokines indicative of immune activation, e.g., IL-2, IFNγ. These simplified assays can mimic important features of the TME relevant to the processes being investigated, while being readily accessible given that they are performed with commercially available reagents and cells.

While the challenges of developing physiologically relevant 3D culture systems to model the cancer immune TME are significant, their potential impact on drug discovery are equally so. Given the significant interest in drug discovery for 3D culture assays to model the cancer immune environment, there is an impressive speed with which new 3D culture platforms are being developed. However, it is a nontrivial challenge to develop 3D culture models that faithfully recapitulate the TME and are robust. We believe that this can be done, especially given the current 3D culture renaissance. The key to integrating 3D culture into drug discovery will be new technologies that facilitate working with 3D cultures in high throughput, greater understanding of the molecular mechanisms underlying immune suppression in the TME, and evolving from more general one-size-fits-all platforms to specialized models tailored to individual questions.

## Data Availability

Data sharing is not applicable to this article as no new data were created or analyzed in this study..

## NOMENCLATURE

CAF: Cancer-associated fibroblast
col-1: Collagen-1
DC: Dendritic cell
ECM: Extracellular matrix
HA: Hyaluronic acid
NK: Natural killer
PEG: Polyethylene glycol
rBM: Reconstituted basement membrane
TAM: Tumor associated macrophage
TCR: T cell receptor
TME: Tumor microenvironment
